# Environmental Investigations of Travel-Associated Legionnaires’ Disease Cases: Timeliness, Microbiological Findings, and Public Health Response

**DOI:** 10.3390/microorganisms14061253

**Published:** 2026-06-02

**Authors:** Antonios Papadakis, Eleftherios Koufakis, Dimosthenis Chochlakis, Anna Psaroulaki

**Affiliations:** 1 Department of Clinical Microbiology and Microbial Pathogenesis, School of Medicine, University of Crete, Voutes—Staurakia, 71110 Heraklion, Greece; surreydimos@hotmail.com; 2 Directorate General of Public Health and Social Welfare, Region of Crete, 71201 Heraklion, Greece; 3 Civil Protection of the Region of Crete, 71201 Heraklion, Greece; elkoufakis@crete.gov.gr; 4 Regional Laboratory of Public Health of Crete, School of Medicine, 70013 Heraklion, Greece

**Keywords:** *Legionella pneumophila*, travel-associated Legionnaires’ disease, environmental investigation, hotel water systems, source investigation, public health response

## Abstract

In Europe, travel-associated Legionnaires’ disease (TALD) cases require timely environmental investigations to support risk assessment, rapid control measures, and prompt reporting of investigation findings to the European Legionnaires’ Disease Surveillance Network (ELDSNet). This study evaluated TALD-related environmental investigations conducted during 2025 and early 2026 in Crete, Greece, following notifications through ELDSNet. Overall, 30 notifications corresponded to 24 unique confirmed TALD cases with illness onset in 2025 and 24 implicated hotels, with some cases involving stays in multiple hotels and Regional Units and clusters identified in some implicated hotels. The investigation framework combined microbiological, physicochemical, and operational data, focusing on delays from symptom onset, notification, sampling, and laboratory reporting. Overall, 516 environmental samples were collected, of which 503 yielded valid analytical results. Among the 503 samples analyzed, *Legionella* spp. were detected at ≥50 colony-forming units per liter (CFU/L) in 127 samples (25.25%). This included 123 samples positive for *L. pneumophila* (24.45%), of which 31 were serogroup 1 (6.16%). Concentrations exceeding the 1000 CFU/L threshold were recorded in 53 samples (10.54%). Operational indicators varied, with median values of 31.0 days for reporting delay (RD), 14.5 days from notification to first sampling (TTF), 47.5 days from symptom onset to first sampling (TDS), and 67.0 days from symptom onset to first laboratory result (OELR). The findings underscore the necessity to document response delays, enhance inspector capacity and cross-regional coordination, and integrate microbiological results with operational indicators. This integration is crucial for facilitating earlier environmental risk assessments, expediting reporting, and implementing more effective TALD public health interventions.

## 1. Introduction

Legionnaires’ disease (LD) is a severe form of pneumonia caused by *Legionella pneumophila*, transmitted through inhalation of contaminated aerosols generated by man-made water systems such as showers, cooling towers, and spa facilities. LD remains a persistent and increasing public health challenge across Europe, with recent European Centre for Disease Prevention and Control (ECDC) surveillance data showing rising notification rates and continued growth in travel-associated reports [1,2]. Approximately one-fifth of these cases are classified as travel-associated Legionnaires’ disease (TALD), linked to exposure in tourist accommodations, including hotels, resorts, and wellness facilities. International surveillance and coordination of TALD events are conducted through the European Legionnaires’ Disease Surveillance Network (ELDSNet) to prevent secondary cases [3,4,5,6,7,8].

In addition, hotel water systems are complex and dynamic, characterized by temperature gradients, stagnation zones, biofilm development, and intermittent use patterns, all of which can sustain colonization by *Legionella* spp., including *L. pneumophila*, even when disinfectant residuals appear adequate [9,10].

From a public health and operational perspective, *L. pneumophila* has particular clinical relevance because it accounts for the great majority of reported LD cases, while serogroup 1 is most frequently associated with severe human disease and travel-associated outbreaks [11,12,13,14]. However, environmental investigations and regulatory interpretation are not limited to *L. pneumophila*, since control measures and exceedance thresholds are commonly based on the detection and concentration of *Legionella* spp. in water systems. Accordingly, effective TALD investigation requires both regulatory assessment of *Legionella* spp. contamination and further characterization of *L. pneumophila* and its serogroups when detected [15,16,17,18].

Previous environmental surveillance and risk-assessment studies conducted in Crete between 2016 and 2025 demonstrated that *Legionella* spp. colonization in tourist accommodations was frequently associated with thermal and chlorine non-compliance, hydraulic dead-legs, seasonal operation, prolonged building closures, and inadequate maintenance practices. These findings showed that routine microbiological testing alone is insufficient to capture the risk of *Legionella* spp. persistence or to reliably predict TALD occurrence. However, although environmental and operational determinants of contamination have been increasingly characterized, the performance of the investigation process itself, particularly in terms of timeliness, inspector capacity, cross-regional coordination, and support for environmental risk assessment and public health response, has received limited systematic evaluation [19,20,21,22,23,24,25,26].

In Greece, TALD notifications follow a standardized pathway coordinated through ELDSNet. Upon international case notification, the Hellenic National Public Health Organization (NPHO) informs the competent Regional Public Health Directorate, which initiates on-site environmental risk assessment and targeted water sampling at the implicated accommodation facility [27,28,29,30].

The added value of this work lies in its focus on the performance of the TALD environmental investigation pathway itself. Specifically, the analysis combines microbiological and physicochemical findings with operational indicators of notification delay, time to first sampling, time to laboratory results, inspector capacity, and cross-regional coordination. In this respect, it shifts emphasis from describing *Legionella* contamination or TALD occurrence alone to evaluating how the timeliness and organization of the public health response influence environmental risk assessment and TALD management.

Environmental investigations triggered by confirmed TALD cases were therefore examined by integrating microbiological, physicochemical, and operational evidence. The analysis documents the delays across the investigation pathway and evaluates how investigation timing, inspector capacity, cross-regional coordination, and water-system conditions affect environmental risk assessment and public health response. These findings are intended to support more timely field investigations, faster reporting of results, and more effective TALD response within the ELDSNet framework.

## 2. Materials and Methods

### 2.1. Study Design and Setting

TALD notifications were communicated through the European Legionnaires’ Disease Surveillance Network (ELDSNet) to the Hellenic National Public Health Organization (NPHO), Directorate of Epidemiological Surveillance and Intervention for Infectious Diseases, Respiratory Infections Unit. NPHO formally notified the competent public health services of the Region of Crete, which coordinated on-site inspections of building water systems, targeted environmental sampling for *Legionella* spp., and verification of corrective actions at the implicated accommodation facilities. The overarching aim of this response pathway was to prevent further cases through timely risk assessment and water quality control.

This study evaluated TALD-related environmental investigations associated with confirmed cases with illness onset in 2025, including investigations conducted during 2025 and early 2026 following delayed notification or operational constraints. All investigations concerned tourist accommodation facilities located on the island of Crete, Greece, one of the most visited regions in the Eastern Mediterranean. The work was carried out by the public health services of the Region of Crete in collaboration with the Regional Public Health Laboratory of Crete.

For analytical and confidentiality purposes, the four Regional Units of Crete were anonymized as Regional Units A–D. Official notifications from NPHO were communicated to all competent services involved in the response, including the Directorate of Public Health and Social Welfare of the relevant Regional Unit, the Regional Directorate of Public Health based at the headquarters of the Region of Crete, and the Directorate General of Public Health and Social Welfare of the Region of Crete. Local inspection and environmental sampling were carried out by the competent Directorate of Public Health and Social Welfare in each Regional Unit. The Regional Directorate of Public Health supported notification management, communication between Regional Units, and reporting, under the administrative guidance of the Directorate General.

The investigation framework was based on the European Technical Guidelines for the Prevention, Control and Investigation of Infections Caused by *Legionella* species [31,32] and national procedures for the management of TALD notifications. Emphasis was placed on the timeliness and effectiveness of field investigations following case notification.

### 2.2. Case Definition and Notification Process

Confirmed TALD cases were identified through ELDSNet and the Hellenic National Public Health Organization (NPHO). The Region of Crete did not perform clinical case confirmation; confirmed TALD notifications were received through the established ELDSNet/NPHO surveillance pathway, based on clinical and laboratory information reported by the diagnosing health-care services. For the purposes of the regional environmental response, eligible investigations were those linked to confirmed TALD notifications with symptom onset during 2025 and at least one implicated accommodation facility located in Crete. Notifications referring to the same individual were consolidated into unique confirmed cases, while each implicated accommodation exposure was retained as a separate hotel-exposure record for investigation purposes. When a case involved stays in more than one hotel or Regional Unit, separate investigation records were created for each case–facility or case–regional episode. No confirmed TALD notification with illness onset in 2025, and an implicated accommodation facility in Crete was excluded from the descriptive case analysis. Environmental samples that could not be analyzed because of excessive background microbial growth were coded as invalid/no result and excluded from microbiological positivity denominators.

Following official notification through the ELDSNet/NPHO pathway, the Regional Public Health Directorate activated the local response team and initiated an on-site environmental investigation at the implicated hotel or accommodation facility. In a limited number of included investigations, official notification and environmental sampling occurred in early 2026, reflecting delayed notification and/or operational constraints after the exposure period.

### 2.3. Sample Collection

Sampling plans were developed individually for each hotel based on architectural schematics and hydraulic layouts, covering cold and hot water distribution systems as well as recreational water facilities. Sampling points included storage tanks, boilers, recirculation loops, risers, distal outlets (taps and showers), and, where present, spa or pool installations.

In total, 516 environmental samples were collected. Of these, 503 were analyzed, while 13 samples could not be analyzed because of excessive background microbial growth. Water samples predominated, while swab samples represented a small proportion of the total sampling set. The sampling strategy focused primarily on distal outlets, particularly room shower water, which accounted for 331/516 collected environmental samples (64.1%). This distribution reflects the targeted and exposure-driven nature of TALD environmental investigations. Sampling prioritized the patient’s room, distal outlets, and aerosol-generating points such as showers, because these locations are most relevant for assessing potential exposure. Therefore, the observed positivity estimates should be interpreted as findings from targeted investigation sampling and not as prevalence estimates for the entire hotel water system. Additional sampling covered boiler outlets and returns, pool- and spa-associated waters, municipal water inlets, kitchen and public taps, water tank outlets, and selected recreational or auxiliary water sources (Table 1).

Sampling procedures followed ISO 19458:2006 [33] for water sampling and ΙSO 56671:2023 Water quality—Sampling Part 1 (Guidance on the design of sampling programs and sampling techniques principles) [34], including selection of representative sampling points, pre-flushing where required, and aseptic handling. Water samples were collected in sterile 1 L bottles containing 20 mg/L sodium thiosulfate to neutralize residual chlorine, transported at 5 ± 3 °C in insulated containers, and processed within 24 h at the Regional Public Health Laboratory of Crete and, when applicable, the Regional Public Health Laboratory of Thessaly.

On-site physicochemical parameters—temperature (°C), free residual chlorine (mg/L), and pH—were measured using calibrated portable instruments. For the assessment and statistical analysis of water-system non-compliance, thresholds of free residual chlorine < 0.2 mg/L, hot-water temperatures < 55 °C, and cold-water temperatures > 25 °C were used to reflect suboptimal control at distal outlets in hotel water systems.

### 2.4. Microbiological and Chemical Analysis

The Regional Laboratory of Public Health of Crete is accredited by the Hellenic Accreditation System (HAS) both for microbiological and chemical analyses carried out in the current study. Moreover, the laboratory participates in external quality assurance schemes such as the External Quality Assessment (EQA) for supporting the surveillance of Legionnaires’ disease, organized by the European Reference laboratory in Public Health on *Legionella* (EURL-PH-LEGI), or the External Quality Assessment (EQA) scheme for supporting the surveillance of Legionnaires’ disease at European level organized by the United Kingdom National External Quality Assessment Service (UKNEQAS).

The detection and quantification of *Legionella* spp. followed ISO 11731:2017 [35]. Water samples were concentrated by membrane filtration (0.2 μm), resuspended in sterile Ringer’s solution (1:40 dilution), and inoculated (200 μL) onto BCYE and GVPC agar media. Aliquots were plated directly, after heat treatment (50 °C for 30 min), and after acid treatment (0.2 mol L^−1^ HCl, pH 2.2). Plates were incubated at 36 ± 2 °C for up to 10 days in humid chambers, with a detection limit of 50 CFU/L.

Presumptive colonies were confirmed by subculture and serotyped into *L. pneumophila* serogroup 1 and serogroups 2–15 using latex agglutination; additional capsular serotyping was applied to differentiate serotypes within serogroups 2–15 when required (Prolex-Lab Diagnostics, Waltham, MA, USA.).

Indicator bacteria were analyzed according to ISO 9308-1:2014 (total coliforms and *E. coli*) [36], ISO 7899-2:2000 (enterococci) [37], and ISO 6222:1999 (total viable counts) [38]. Physicochemical analyses followed the Standard Methods for the Examination of Water and Wastewater (APHA, 24th edition) and HACH-validated in-house procedures, including calcium, magnesium, nitrates, nitrites, ammonium, sulfates, chlorides, pH, conductivity, total hardness, alkalinity, and turbidity. In situ measurements (temperature, chlorine, pH, conductivity) were recorded at each sampling point after 2 min of flow [39].

### 2.5. Operational Indicators and Statistical Analysis

To assess the timeliness and field performance of TALD-related environmental investigations, operational and microbiological indicators were calculated for each investigation. The indicators covered reporting delay, environmental response time, time from symptom onset to sampling, laboratory result timelines, field-team sampling productivity, service-adjusted sampling efficiency, and *L. pneumophila* detection outcomes. These operational indicators were designed as descriptive performance measures of the TALD environmental investigation pathway. They were not intended to function as externally validated epidemiological risk scores or predictors of infection risk. Their purpose was to summarize response timeliness, sampling effort, laboratory-result availability, and service-level capacity in a standardized manner across investigations. The selection of timing indicators was guided by the investigation framework developed by the European Working Group for *Legionella* Infections (EWGLI) and subsequently incorporated into the European Legionnaires’ Disease Surveillance Network (ELDSNet), as well as by the operational requirement for timely environmental assessment after TALD notification [32]. The 14-day reference used for time-to-first sampling and efficiency calculations was applied as an operational benchmark reflecting the EWGLI/ELDSNet two-week timeframe for preliminary risk assessment and initiation of control measures after an alert, rather than as a strict microbiological sampling deadline. Composite indicators, including ER, SPD, and SASE, were used only to support internal comparison of field-response performance between investigations and Regional Units and were interpreted descriptively. Definitions, calculation bases, units, and public-health rationale for all operational indicators are summarized in Table 2.

At the case/facility level, the following indicators were computed:

Reporting Delay (RD, days):

(1) RD=Notification Date−Onset Date 

This indicator expresses the interval between the reported symptom onset date and receipt of the official notification by the Regional Public Health Directorate of the Region of Crete from NPHO. It captured the delay up to activation of the regional environmental response, rather than the full international notification pathway. The date on which ELDSNet/ECDC first notified NPHO was not available and was treated as an unmeasured component of the surveillance pathway and a study limitation.

Time-to-First Sampling (TTF, days):

(2) TTF=First Sampling Date−Notification Date 

This indicator measured the operational response time of the regional public health service, defined as the interval between receipt of the official NPHO notification by the Regional Public Health Directorate and the first environmental sampling visit at the implicated accommodation facility. It was interpreted relative to the 14-day operational benchmark described above [29].

Time from Symptom Onset to First Sampling (TDS, days):

(3) $$\begin{array}{cccc} & TDS=First\;Sampling\;Date-Reported\;Symptom\;Onset\;Date & & \end{array}$$

This indicator measured the total interval from the reported symptom onset date to the first environmental sampling visit, incorporating both the pre-notification delay and the subsequent field response time.

Onset-to-First Laboratory Result (OELR, days):

(4) $$\begin{array}{cccc} & OELR=First\;Laboratory\;Result\;Date-Reported\;Symptom\;Onset\;Date & & \end{array}$$

This indicator measured the total interval from the reported symptom onset date to the availability of the first laboratory result from environmental sampling.

Notification-to-First Laboratory Result (NELR, days):

(5) $$\begin{array}{cccc} & NELR=First\;Laboratory\;Result\;Date-Notification\;Date & & \end{array}$$

This indicator measured the interval from receipt of the official notification by the Regional Public Health Directorate to the availability of the first laboratory result from environmental sampling.

Efficiency Ratio (ER):

(6) $$ER=\left(\frac{14}{TTF}\right)$$

This indicator expresses the timeliness of the field response relative to the 14-day reference target for initiating environmental sampling after notification. Values greater than 1 indicated sampling faster than the 14-day target, whereas values below 1 indicated delayed initiation of sampling. ER was used as a descriptive measure of whether the first sampling was initiated faster or slower than the 14-day operational reference target; it was not interpreted as a validated epidemiological risk index.

Sampling Productivity (SPD):

(7) $$SPD=\frac{\mathrm{Samples}\;\mathrm{collected}\;\mathrm{during}\;\mathrm{the}\;\mathrm{first}\;\mathrm{sampling}\;\mathrm{visit}}{TTF}$$

This indicator expresses the sampling output of the field team in relation to the time required to initiate the first environmental sampling after notification. It was calculated as the number of samples collected during the first sampling visit divided by TTF. Higher values indicated greater sampling output within a shorter response interval.

Service-Adjusted Sampling Efficiency (SASE):

(8) $$SASE=\frac{\mathrm{Total}\;\mathrm{Samples}}{TTF\times \mathrm{Inspectors}\;\mathrm{Available}\;\mathrm{in}\;\mathrm{Service}}$$

This indicator integrated timeliness, sampling effort, and staffing capacity. Because several TALD cases involved stays in more than one hotel and in more than one Regional Unit, the same framework was applied at the regional level, using separate case–Region records, to allow comparison between services with different inspector capacity. SASE was introduced as a descriptive, capacity-adjusted operational indicator for internal comparison of field-response performance between Regional Units with different inspector availability. No external benchmark or validated threshold exists for SASE; therefore, the reported values should be interpreted only in relative terms—higher values indicate greater sampling output per inspector-day relative to the notification-to-sampling interval—and not as validated epidemiological risk scores or absolute performance standards.

Positivity Rate (%):

(9) $$\begin{array}{cccc} & Positivity\;Rate=\frac{L.\;pneumophila\text{-}positive\;analyzed\;samples\;(\geq 50\;\mathrm{CFU}/L)}{Total\;analyzed\;samples}\times 100 & & \end{array}$$

This indicator expressed the proportion of analyzed samples positive for *L. pneumophila* at or above the analytical detection limit of 50 CFU/L. Samples coded as “no result” because of excessive background microbial growth were excluded from the denominator. Samples with *L. pneumophila* concentrations > 1000 CFU/L were analyzed separately as threshold-exceeding samples.

Threshold Exceedance Rate (%):

(10) $$\begin{array}{cccc} & Threshold\;Exceedance\;Rate=\frac{L.\;pneumophila\;samples>1000\;\mathrm{CFU}/L}{Total\;analyzed\;samples}\times 100 & & \end{array}$$

This indicator expressed the proportion of analyzed samples exceeding the >1000 CFU/L threshold for *L. pneumophila*. It was analyzed separately from overall positivity because low-level detection and threshold exceedance have different operational and public health interpretations.

SG1 Proportion (%):

(11) $$\begin{array}{cccc} & SG1\;Proportion=\frac{SG1}{SG1+SG2-15}\times 100 & & \end{array}$$

This indicator expressed the proportion of *L. pneumophila* serogroup 1 among serotyped *L. pneumophila*–positive samples. SG1 was summarized separately because of its clinical and epidemiological relevance in LD investigations and source-attribution assessment.

Regional Notification Delay (RND, days):

(12) RND=Regional Notification Date−Reported Symptom Onset Date 

This indicator measured the interval between the reported symptom onset date and receipt of the official NPHO notification by the competent Regional Public Health Directorate for the specific Regional Unit involved in the investigation. As with RD, this indicator did not capture the date on which ELDSNet/ECDC first notified NPHO, because that information was not available.

Regional Time-to-First Sampling $\left(TTF_{r},\;\mathrm{days}\right)$:

(13) $$TTF_{r}=\mathrm{First}\;\mathrm{Sampling}\;\mathrm{Date}-\mathrm{Regional}\;\mathrm{Notification}\;\mathrm{Date}$$

This indicator measured the operational response time of the competent Regional Public Health Directorate for the specific Regional Unit involved in the investigation. It was defined as the interval between receipt of the official notification by that service and the first environmental sampling visit conducted in the corresponding Regional Unit.

Regional Efficiency Ratio $\left(ER_{r}\right)$:

(14) $$ER_{r}=\frac{14}{TTF_{r}}$$

This indicator expresses the timeliness of the regional field response relative to the 14-day reference target for initiating environmental sampling after notification. Values greater than 1 indicated sampling faster than the 14-day target, whereas values below 1 indicated delayed initiation of sampling in the corresponding Regional Unit.

Regional Service-Adjusted Sampling Efficiency $\left(SASE_{r}\right)$:

(15) $$SASE_{r}=\frac{\mathrm{Samples}\;\mathrm{collected}\;\mathrm{during}\;\mathrm{the}\;\mathrm{first}\;\mathrm{sampling}\;\mathrm{visit}\;\mathrm{in}\;\mathrm{the}\;\mathrm{Regional}\;\mathrm{Unit}}{TTF_{r}\times \mathrm{Inspectors}\;\mathrm{Available}\;\mathrm{in}\;\mathrm{Service}}$$

To account for unequal inspector pools across Regional Units, capacity-normalized regional indicators were derived:

(16) $$cTTF_{r}=TTF_{r}\times \frac{2}{\mathrm{Inspectors}\;\mathrm{Available}\;\mathrm{in}\;\mathrm{Service}},cER_{r}=\frac{14}{cTTF_{r}},cSASE_{r}=\frac{\mathrm{Total}\;\mathrm{Samples}}{cTTF_{r}\times 2}$$

These indicators normalized regional response performance to a reference two-inspector capacity, allowing comparison between Regional Units with different available inspector pools.

Finally, a Service Load Ratio (SLR) was computed as:

(17) $$SLR=\frac{2}{\mathrm{Inspectors}\;\mathrm{Available}\;\mathrm{in}\;\mathrm{Service}}$$

This indicator expressed how close each Regional Unit was to the reference two-inspector field deployment.

Required staffing per Regional Unit was estimated with a simple capacity model:

(18) $$n^{*}=\lceil \frac{R\times w}{A\times u^{*}}\rceil$$

where R is the case arrival rate (cases per chosen period), w the median inspector-days to first sampling (ID–TTF) per case, A the available inspector-days per inspector in the same period, and $u^{*}\;$the target utilization.

Each environmental investigation was linked to one or more travel-associated TALD cases notified from the same hotel within the same month. When multiple TALD cases were associated with the same facility and sampling visit, the inspection and sampling round was recorded once, while the number of related cases was captured separately. Environmental data were therefore analyzed at the hotel–sampling-round level rather than at the individual-case level.

When a TALD case involved stays in more than one hotel or more than one Regional Unit, separate investigation records were created for each case–facility or case–regional episode. Operational indicators were calculated at the level of environmental investigations, whereas descriptive epidemiology was based on 24 unique confirmed TALD cases with illness onset in 2025.

### 2.6. Statistical Analysis

All statistical analyses were performed using IBM SPSS Statistics v30.0 (IBM Corp., Armonk, NY, USA), Epi Info v7.2.7.0 (Centers for Disease Control and Prevention, Atlanta, GA, USA), and the MedCalc Relative Risk Calculator Version 23.4.2 (MedCalc Software Ltd., Ostend, Belgium).

Analyses were explicitly separated according to the relevant analytical level. Descriptive epidemiology was performed at the unique-case level, operational indicators were summarized at the hotel–sampling-round level, and microbiological associations were examined at the sample level.

Descriptive analyses included frequencies, proportions, medians, interquartile ranges, and 95% confidence intervals. Proportions were summarized using Wilson score 95% confidence intervals, while Clopper–Pearson exact 95% confidence intervals were applied for small sample sizes where appropriate.

Associations between categorical variables were assessed using the chi-square test or Fisher’s exact test, as appropriate. These bivariate analyses were interpreted as exploratory and descriptive. To evaluate the associations between facility/water-system characteristics and *L. pneumophila* detection while adjusting for potential confounders, a multivariable logistic regression was performed using Epi Info v7. The model included free chlorine non-compliance (<0.2 mg/L), hot-water temperature < 55 °C, cold-water temperature > 25 °C, hotel rating < 4 stars, and hotel size > 200 beds. Adjusted odds ratios (aORs) with 95% confidence intervals were reported. Because multiple samples originated from the same hotels, and because available software constraints and the limited number of implicated hotels prevented the reliable use of mixed-effects models to fully account for data non-independence, adjusted estimates were interpreted cautiously regarding their precision and were not considered causal effects. As a sensitivity analysis, we re-ran the logistic regression model restricted to one randomly selected sample per hotel to assess the potential impact of within-hotel clustering on the main estimates. The direction and magnitude of associations were consistent with the primary analysis, supporting the robustness of the reported findings despite the acknowledged non-independence.

Analyses focused on *L. pneumophila* positivity or threshold exceedance were reported separately where applicable.

Spearman’s rank correlation was used to explore associations between operational indicators where applicable. Statistical significance was defined as *p* < 0.05, while highly significant results were defined as *p* < 0.0001. To evaluate the robustness of our multivariable logistic regression findings against potential unmeasured confounding, we calculated E-values for the significant environmental risk factors. The E-value defines the minimum strength of association that an unmeasured confounder would need to have with both the exposure and the outcome to explain away the observed aORs [40,41,42].

Samples for which microbiological analysis could not be performed because of excessive background microbial growth were coded as “no result”, treated as missing data, and excluded from primary positivity analyses. The frequency of these samples was described separately.

## 3. Results

### 3.1. Descriptive Epidemiology of the Confirmed Cases

**Figure 1:**
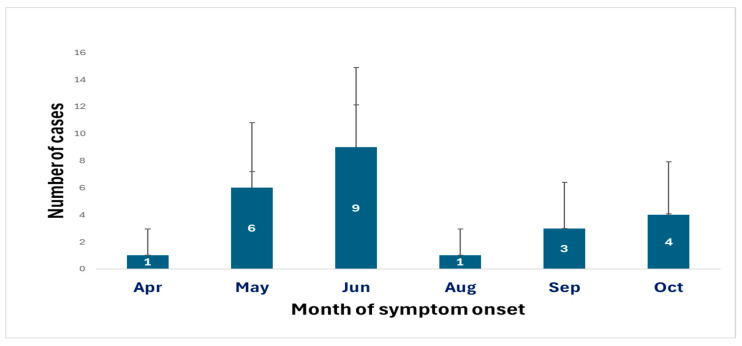
Monthly epidemic curve of unique confirmed travel-associated Legionnaires’ disease (TALD) cases by symptom onset month in Crete, 2025. Error bars represent approximate 95% Poisson confidence intervals.

**Figure 2:**
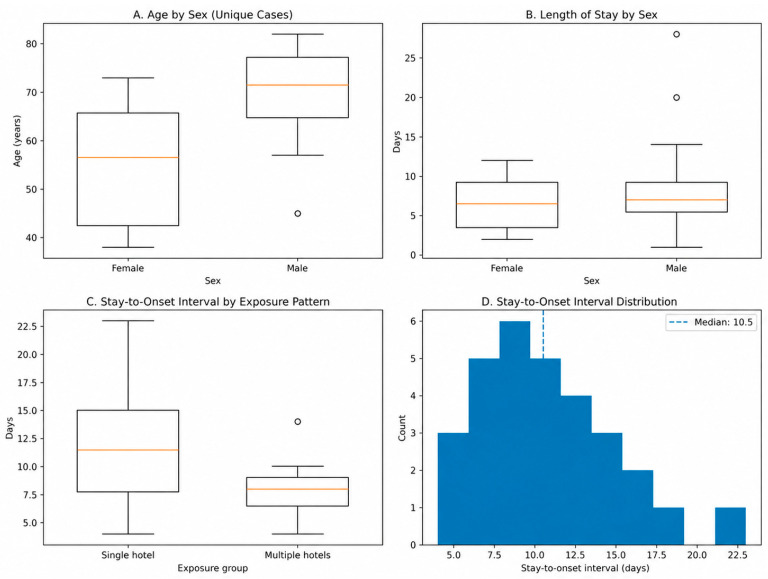
Demographic and exposure-related characteristics of TALD cases.

During the 2025 surveillance period, 24 unique confirmed cases of TALD with illness onset in 2025 were recorded among international travelers who stayed in implicated accommodation facilities across Crete. Overall, 30 notifications were received, corresponding to 24 implicated hotels and 30 hotel-exposure records. Four cases involved stays in more than one hotel, and three of these also involved more than one Regional Unit, requiring coordinated investigations between the respective public health services. Two clusters were identified among the implicated hotels: one hotel was associated with six TALD cases, and another hotel with two TALD cases. The median age of affected cases was 68 years (range: 38–82), with a predominance of males (16 males, 8 females). Most cases were residents of Germany (*n* = 7) or the United Kingdom and its constituent nations (*n* = 7), followed by France (*n* = 4) and Norway (*n* = 2), with additional sporadic cases from the USA, Belgium, Finland, and Slovakia (*n* = 1 each). The urinary antigen test remained the predominant diagnostic method (20/24, 83.33%), while 4 cases (16.67%) were confirmed by PCR. Clinical outcomes were favorable in most instances, with 19 cases recovering, four cases remaining with unknown outcomes, and one fatality recorded in a 67-year-old male traveler. Across the hotel-exposure records, the median interval from the first recorded hotel-stay date to symptom onset was 10.5 days (IQR: 7.25–13; range: 4–23), while the median length of stay per hotel was 7 days (IQR: 4.25–9.75; range: 1–28). This variable should not be interpreted as the biological incubation period in all cases, because some travelers had prolonged stays or multiple accommodation exposures, and infection may have occurred later during the stay within the accepted TALD exposure window. The median delay between reported symptom onset and official notification to the Regional Public Health Directorate was 31.5 days (IQR: 25.5–49; range: 11–118), indicating considerable lag between illness onset and activation of the regional environmental response. The temporal distribution of onset dates is shown in the epidemic curve (Figure 1), which demonstrates a concentration of TALD cases during the tourism season, with Poisson confidence intervals illustrating the statistical variability in monthly counts. A composite descriptive figure (Figure 2) further summarizes these epidemiological features, illustrating the age–sex distribution, length of stay, and the stay-to-onset interval distributions stratified by exposure profiles. Together, Figure 1 and Figure 2 provide an integrated overview of the demographic, temporal, and exposure-related characteristics of TALD cases during the 2025 surveillance period.

### 3.2. Operational and Microbiological Performance

During 2025 and early 2026, TALD-related environmental investigations were conducted across four anonymized Regional Units of Crete (A–D). For operational and microbiological performance analysis, results were summarized at the hotel–sampling-round level to avoid double-counting sampling visits linked to more than one TALD case. Overall, 20 completed hotel–sampling-round investigations with recorded sample counts were included in the main performance analysis. Across the 20 completed hotel–sampling-round investigations, 305 samples were collected during the corresponding first sampling visits. *L. pneumophila* was detected at ≥50 CFU/L in 89/305 samples (29.18%), while 40/305 samples (13.11%) exceeded 1000 CFU/L. The full distribution of operational and microbiological performance indicators is summarized in Table 3. 

The exploratory comparison between the reporting-delay category and environmental *L. pneumophila* detection is shown in Table 4.

Although reporting delays were substantial, no statistically significant association was observed between reporting-delay category and environmental *L. pneumophila* detection. Therefore, reporting delay was not confirmed as a statistical predictor of environmental positivity in this dataset. This null result should be interpreted with caution, given the small number of investigations with RD ≤ 14 days (*n* = 2), which severely limits statistical power to detect a true association. Additionally, the persistence of *Legionella* in biofilm within hotel water systems may allow detection even after delayed sampling, which could explain the absence of a clear association between reporting delay and culture positivity in this dataset.

### 3.3. Cross-Regional Case Investigations

Three TALD cases involved stays in more than one Regional Unit, necessitating coordinated environmental investigations between the competent local public health authorities. These cross-regional investigations generated eight regional investigation records across four Regional Units (A–D), of which six had completed first sampling at the time of analysis, and two were still pending. The completed cross-regional investigations demonstrated marked heterogeneity in notification timing, operational responsiveness, and service-level capacity (Table 5).

### 3.4. Inspector Capacity for TALD Field Investigations After Case Notification, by Regional Unit

Using the observed regional median time-to-first sampling (TTF_r_) and the operational target of ≤14 days, we estimated the inspector capacity required specifically for TALD environmental investigations and first-round water sampling following case notification (Table 6). These estimates reflect task-specific capacity for TALD response only and do not represent total staffing needs for other public health duties. As detailed in Table 6, Regional Unit D met the timeliness target at its current staffing level, and Regional Unit C demonstrated potential surplus capacity. In contrast, Regional Units A and B exhibited prolonged response times, requiring additional estimated inspector capacity.

However, these estimates should be interpreted as task-specific planning indicators, since delays may also reflect notification routing, seasonal constraints, hotel availability, and pre-scheduled sampling capacity rather than inspector numbers alone.

**Figure 3:**
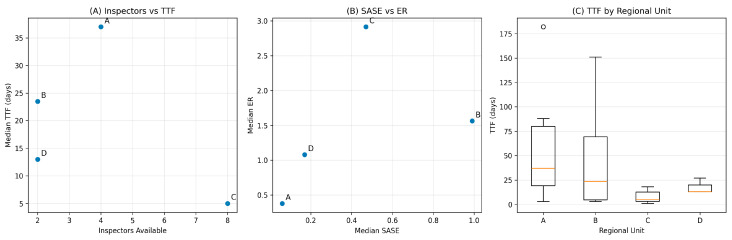
Inspector capacity and operational performance by anonymized Regional Unit (A–D), Crete, 2025.

**Figure 4:**
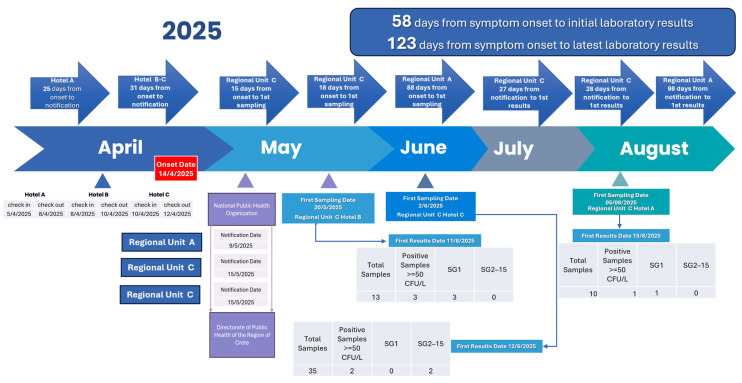
Schematic timeline of a representative TALD investigation (Crete, 2025), distinguishing notification delays from the environmental response timeframe.

Associations between operational indicators are shown in Figure 3. Inspector availability was inversely related to TTF_r_, while higher service-adjusted sampling efficiency (SASE_r_) was associated with higher regional efficiency ratios (ER_r_) across Regional Units. The distribution of TTF_r_ by Regional Unit further illustrates inter-regional heterogeneity in operational responsiveness. To further illustrate how these delays accumulated during the investigation pathway, a schematic timeline of a representative TALD investigation is shown in Figure 4. The timeline presents the sequence from hotel stay and symptom onset to official notification, first environmental sampling, and availability of the first laboratory results, highlighting the relative contribution of pre-notification delay and post-notification environmental response time.

### 3.5. Timeliness and Environmental Investigation Context of TALD-Associated Hotels

In a subset of hotels linked to TALD cases, repeated environmental investigations were undertaken following case notification. In one facility, a single TALD case was identified in 2024, followed by two additional cases in 2025 (three cases in total). All water samples collected prior to notification of the first case were negative for *Legionella*. Subsequent sampling rounds conducted after the first and second cases yielded several *L. pneumophila* positive samples, followed by a fully negative round later in the season. Through these investigations, free chlorine concentrations remained within the recommended range (≥0.2 mg/L), whereas hot water temperatures were below 55 °C in 14 of 40 measurements (35.0%) and cold-water temperatures exceeded 25 °C in 18 of 58 outlets (31.0%), indicating suboptimal thermal control despite adequate disinfectant residuals.

A second hotel experienced a larger TALD cluster, with two cases in 2023 and six additional cases in 2025 (eight cases in total). Four environmental investigations were conducted during 2025 (14 April, 11 June, 23 June, and 10 July). Free chlorine concentrations were consistently ≥0.2 mg/L in all rounds, while temperature control again remained suboptimal, with repeated exceedances of recommended thresholds for both hot and cold water. Overall, 14 of 100 water samples (14.0%) were culture-positive for *L. pneumophila*, including serogroup 1 (10%) and serogroup 3 (4%) isolates. All TALD cases in both hotels were diagnosed by urinary antigen testing.

Across TALD-associated hotels, the interval between symptom onset and notification of local public health authorities (reporting delay, RD) ranged from 11 to 32 days (median 26 days). The time from onset to the first environmental sampling visit (TDS) ranged from 12 to 40 days (median 31 days). By contrast, the delay between notification and initiation of environmental sampling (time-to-first sampling, TTF) was markedly shorter, ranging from 1 to 13 days (median 4 days). In all investigations, two inspectors per visit were deployed from a pool of eight available inspectors, indicating that the majority of the overall delay occurred before notification, rather than during the organization of environmental investigations once authorities had been informed.

The sequence of key actions, including hotel stay, symptom onset, notification, environmental sampling rounds, and availability of first laboratory results, is illustrated schematically in Figure 4, highlighting the cumulative effect of notification delays on the overall investigation timeline. 

### 3.6. Facility and Water-System Factors Associated with L. pneumophila Positivity

In the multivariable logistic regression analysis (Table 7), free chlorine concentrations < 0.2 mg/L, hot water temperatures < 55 °C, and hotel ratings < 4 stars were independently associated with increased odds of *L. pneumophila* positivity, whereas larger establishments (>200 beds) were protective. In contrast, cold water temperatures > 25 °C did not show a significant association with detection (aOR 0.56, 95% CI 0.24–1.33; *p* = 0.189). Sensitivity analysis supported the robustness of these findings, indicating that substantial unmeasured confounding would be required to negate the observed associations for hot water temperature and hotel rating.

Circulation-related parameters, including boiler outlet temperature < 60 °C and elevated hot-water return temperature (>10 °C), were not significantly associated with positivity. These analyses were limited by sparse data and wide confidence intervals and were therefore interpreted cautiously using Fisher’s exact test.

Overall, 13 samples from five hotels could not be analyzed because excessive background microbial growth prevented reliable *Legionella* detection or enumeration. These samples were coded as invalid/no result and excluded from microbiological positivity denominators.

### 3.7. L. pneumophila Serogroup Distribution Across Sampling Rounds

Overall, *L. pneumophila* was detected in 123/503 analyzed samples (24.45%; 95% CI: 20.90–28.39%), while 380/503 samples were negative (75.55%; 95% CI: 71.61–79.10%). Non-*pneumophila Legionella* spp. accounted for only 4/503 analyzed samples (0.80%), corresponding to 4/127 *Legionella* spp. positive samples (3.15%). Thirteen additional samples could not be analyzed because of excessive background microbial growth and were recorded as “not done”.

The serogroup distribution of *L. pneumophila* in hotel water samples showed that serogroup 1 (SG1) was detected in 31/503 analyzed samples (6.16%; 95% CI: 4.26–8.40%), while 472/503 samples were SG1-negative (93.84%). SG1 detections were observed across a range of colony-forming unit (CFU) counts, including low-level detections at 50 CFU/L and higher concentrations reaching 10,001 CFU/L.

Serogroups 2–15 (SG2–15) were detected in 95 samples, corresponding to 18.9% of all analyzed samples (95/503) and 77.2% of *L. pneumophila* positive samples (95/123). Detected SG2–15 concentrations ranged from 50 to 11,650 CFU/L, with 23/95 SG2–15-positive samples (24.2%) recorded at 50 CFU/L and 13/95 samples (13.7%) recorded above 10,000 CFU/L.

The distribution of *L. pneumophila* serogroups varied across the three sampling rounds (Table 8). The distribution of *L. pneumophila* serogroups varied across the three sampling rounds (Table 8). Overall, SG2–15, SG1, SG3, SG6, and SG8 were detected across multiple sampling rounds, whereas SG7, SG9, and SG15 appeared sporadically.

Overall, these findings indicate that *L. pneumophila* accounted for nearly all *Legionella-positive* results, with SG2–15 representing the predominant serogroup category among *L. pneumophila-positive* samples. SG1 was less frequent overall but remained epidemiologically relevant because of its recognized association with human LD and source-attribution assessment.

### 3.8. Indicator Bacteriology and Physicochemical Findings

Indicator bacteriology and free chlorine compliance for the selected water samples are detailed in Table 9. Overall, these findings indicate that microbiological non-compliance was driven primarily by elevated total coliform counts, whereas *fecal* indicator bacteria were rare and free chlorine deficiencies were limited to a small subset of samples.

**Figure 5:**
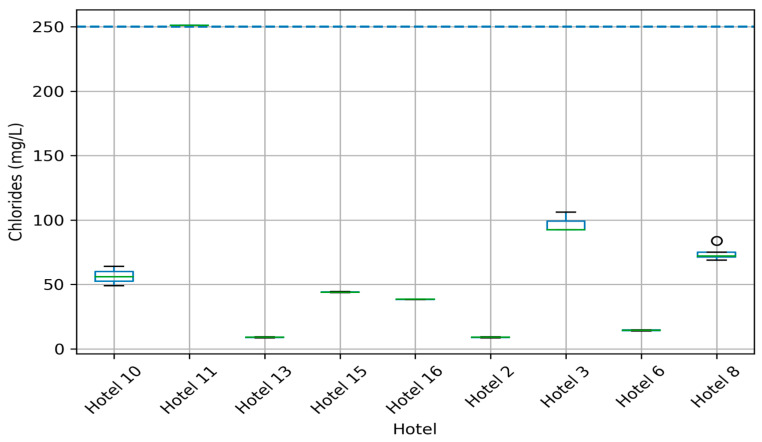
Distribution of chloride concentrations across sampled hotels. Boxplots show chloride concentrations (mg/L) by hotel. The dashed horizontal line indicates the parametric guideline value of 250 mg/L. Elevated chloride levels were observed exclusively in hotel 11, whereas all other hotels remained below the guideline threshold.

Across the analyzed water samples, pH values were consistently within acceptable limits, with no instances of non-compliance recorded. Electrical conductivity showed moderate variability between sampling points but remained within regulatory thresholds in all samples. Sulfate concentrations were also compliant throughout the dataset, despite higher values being observed in selected tank outlets and garden taps. Nitrite concentrations were uniformly low (≈0.05–0.06 mg/L) and did not exceed guideline values in any sample. In contrast, chloride concentrations were the most frequently non-compliant chemical parameter. Exceedances were observed primarily in samples from Hotel 11, particularly during repeated samplings in June and September, affecting municipal water inlets, room taps, showers, and fountain water (Figure 5). Outside this facility, chloride levels remained consistently within acceptable limits across hotels and sample types. Overall, chemical non-compliance was limited and parameter-specific, being driven almost exclusively by elevated chloride concentrations at a single hotel, while pH, conductivity, sulfates, and nitrites showed stable compliance across the study period.

The environmental investigations revealed a significant variability in physicochemical data completeness, which was closely associated with regional service capacity and water-system type. In hot water distribution systems (*n* = 216), temperature recording was highly consistent, with omissions occurring in only 3.24% of the collected samples. These omissions were strictly localized in the most operationally burdened jurisdictions, as Regional Unit A recorded a 13.95% omission rate and Regional Unit B a 2.78% rate, while Regional Units C and D achieved 100% documentation. Among documented measurements, 70.83% of hot water samples were thermally non-compliant (below 55 °C), with Regional Unit D reaching a peak non-compliance rate of 84.72%.

In cold water distribution systems (*n* = 273), a hierarchical systemic gap in documentation was observed. Island-wide, pH measurements were missing in 53.48% of samples, followed by residual chlorine in 26.37% and temperature in 15.38%. Data completeness was poorest in Regional Unit A, where omissions reached 83.87% for pH, 41.94% for chlorine, and 38.71% for temperature. Regional Unit B followed a similar trend with 79.63% missing pH and 40.74% missing chlorine, although it achieved 90.74% temperature documentation. In contrast, the higher-capacity Regional Units C and D showed improved performance; Unit C recorded 93.06% of temperatures, and Unit D achieved near-universal documentation for chlorine (98.82%) and temperature (90.59%). However, pH omissions remained significant even in these units, at 36.11% in Unit C and 29.41% in Unit D. Regarding compliance, 42.86% of cold-water samples exceeded the 25 °C threshold, while inadequate residual chlorine below 0.2 mg/L was identified in 17.58% of documented cases.

## 4. Discussion

This study evaluated TALD-related environmental investigations in a major Mediterranean tourist destination by integrating microbiological findings, physicochemical water-system data, and operational response indicators. Three main messages emerge from the analysis: notification and sampling delays affect the interpretation of environmental findings; microbiological results should be integrated with water-system and operational indicators; and coordinated regional response capacity is essential for TALD investigations. Overall, the findings show that TALD environmental investigations cannot be interpreted through culture results alone; negative or discordant microbiological results must be assessed in relation to notification timing, sampling timing, possible prior corrective actions, hotel water-system conditions, and the broader epidemiological context.

A key operational finding was that much of the delay occurred before the regional public health services were activated. Once the Regional Public Health Directorate received the official notification, environmental investigations were generally organized within a shorter timeframe. This distinction is important because delayed notification may substantially weaken the interpretive value of environmental cultures. By the time sampling is performed, the implicated water system may no longer reflect the conditions present during the traveler’s exposure period. This is particularly relevant in hotel settings, where managers may initiate flushing, hyperchlorination, thermal disinfection, cleaning of outlets, or other corrective actions immediately after being informed of a possible TALD event. Under these circumstances, negative cultures may reflect the timing of sampling and recent remediation rather than the true absence of prior colonization. Accordingly, reporting and sampling intervals should be treated as interpretive variables within TALD investigations, rather than as purely administrative performance indicators [43,44].

The microbiological results also underline the value of repeated sampling. The serogroup profile was not static across sampling rounds, with SG2–15 representing the predominant *L. pneumophila* category and several non-SG1 serogroups recurring over time. This pattern is compatible with the known ecology of *Legionella* in engineered water systems, where biofilm, stagnation, temperature gradients, and intermittent hydraulic conditions can support spatially heterogeneous and temporally variable colonization [5,45,46]. The reduction or disappearance of some serogroups in later rounds should therefore be interpreted cautiously. It may reflect the effect of control measures, sampling variability, intermittent detachment from biofilms, or incomplete serotyping, rather than definitive eradication.

Although SG1 was less frequent than SG2–15 in the environmental samples, it remains epidemiologically important because of its strong association with human LD and the widespread use of urinary antigen testing, which primarily detects *L. pneumophila* serogroup 1. This has direct implications for environmental risk assessment and source investigation. In this context, an absence of SG1 in environmental samples should be interpreted cautiously, particularly when sampling was delayed or when control measures may have altered the system before official investigation. This limitation is well recognized in LD investigations, where clinical–environmental strain comparison remains essential for robust source confirmation [47,48,49,50,51].

The predominance of *L. pneumophila* among positive results supports its central role in TALD risk assessment. However, the detection of a small number of non-pneumophila *Legionella* spp. samples also supports maintaining a broader environmental surveillance perspective. From a clinical perspective, *L. pneumophila* and its serogroups are most relevant for interpreting TALD cases [52,53,54,55,56]. From a water-safety and regulatory perspective, however, *Legionella* spp. detection remains important because it indicates colonization of the water system and the need for control measures. This dual interpretation is consistent with European and international approaches to *Legionella* prevention, which combine system-level control of *Legionella* contamination with further characterization of clinically relevant *L. pneumophila* isolates when detected [57,58,59,60,61].

The associations between water-system characteristics and *Legionella* detection were consistent with established principles of control. The adjusted analysis strengthened the interpretation that free chlorine non-compliance and suboptimal hot-water temperature were important operational markers associated with *L. pneumophila* detection. The robustness of the association between suboptimal hot water temperature and *L. pneumophila* detection is further highlighted by the high E-value (10.42). This suggests that any unmeasured confounder, such as biofilm complexity or the presence of specific protozoa, would need to have a very strong association (exceeding 10-fold) with both the exposure and the outcome to explain away the observed effect. This reinforces the role of temperature as a dominant and resilient independent driver of colonization in these hotel water systems.

However, the numerical estimates reported in this study should be interpreted in relation to the targeted investigation design, repeated sampling context, and uncertainty reflected by the confidence intervals. Because multiple environmental samples originated from the same accommodation facilities, residual non-independence from within-hotel clustering may have affected the precision of the effect estimates. Therefore, the reported aORs should be viewed as adjusted exploratory estimates rather than prevalence estimates or definitive causal effects. These findings agree with previous studies from Greek hotel water systems, where *Legionella* colonization was associated with inadequate temperature control, insufficient disinfectant residuals, seasonal operation, and building-level water-system characteristics [20,21,22,23,25,26,62,63,64,65,66,67,68,69]. The lack of a clear association for some circulation-related parameters should not be interpreted as evidence of no effect; rather, these variables were limited by sparse data and incomplete measurement across systems.

The structural understaffing identified across the study area is further underscored when evaluated against international public health benchmarks. According to WHO standards, a minimum ratio of one public health inspector per 10,000 residents is recommended to ensure effective environmental health surveillance and response capacity [40,70]. Based on the 2021 Hellenic Statistical Authority (ELSTAT) census data, all Regional Units in Crete operate with a staffing deficit of approximately 75% [71]. This deficit is most acute when considering both general public health needs and task-specific requirements for travel-associated Legionnaires’ disease investigations. For instance, Regional Unit A would require 16 inspectors by international standards and 11 based on the study’s capacity model to achieve a timely response, yet it operates with only 4. Regional Unit B, requiring 9 inspectors by international standards and 4 by the capacity model, is currently staffed by only 2. Similarly, Regional Unit C employs 8 inspectors despite a population-based requirement for 31, although its operational efficiency allows it to exceed the task-specific requirement of 3 inspectors derived from the model. Finally, Regional Unit D operates with 2 inspectors, meeting its task-specific requirement of 2 but falling short of the 8 recommended by international benchmarks. This chronic staffing deficit across all jurisdictions creates severe operational pressure, particularly during periods of high demand, explaining why field teams are often forced to prioritize rapid microbiological sampling over comprehensive physicochemical documentation. These results indicate that the observed delays and data gaps are not merely localized inefficiencies but are deeply rooted in a workforce capacity that falls significantly short of established global safety standards.

The two cluster-associated hotels illustrate the practical difficulty of environmental risk assessment and source investigation in TALD. Repeated investigations showed intermittent *L. pneumophila* detection, suboptimal thermal control despite adequate free chlorine, and differences between clinical diagnostic evidence and environmental serogroup findings. In one hotel, the environmental findings did not fully match the serogroup most strongly implied by urinary antigen testing, while in the other, SG1 was detected in environmental samples, but clinical isolates were not available for strain comparison. These examples support classifying such hotels as plausible exposure sites rather than confirmed sources unless molecular evidence is available. They also demonstrate how repeated sampling and integration of clinical, microbiological, and operational information can prevent premature exclusion or confirmation of a suspected exposure site [72]. The maximum stay-to-onset interval of 23 days should not be interpreted as a 23-day biological incubation period. Rather, it reflects the interval from the first recorded hotel-stay date to symptom onset in travelers with prolonged or multiple accommodation exposures; the actual exposure could have occurred later during the stay and within the accepted TALD exposure window. Similarly, reporting delay should be interpreted as an operational barrier to timely environmental risk assessment and reporting, rather than as a confirmed predictor of culture positivity, since no statistically significant association was observed between reporting-delay category and *L. pneumophila* detection in this dataset.

The cross-regional investigations highlight an additional operational challenge. When a traveler stayed in more than one accommodation facility and more than one Regional Unit, the investigation could easily become fragmented if handled only at the subregional level. Central coordination by the Regional Public Health Directorate helps preserve the epidemiological linkage between exposures, prevents duplicate or disconnected records, and supports a more coherent interpretation of hotel-level findings. In this setting, coordination is not simply an administrative issue; it directly affects the quality of environmental risk assessment and source investigation, as well as the consistency of the TALD response.

Indicator bacteriology and physicochemical findings provided useful complementary information. Total coliform non-compliance in selected samples, in the absence of widespread fecal indicators, suggests local regrowth, stagnation, or distribution-system management issues rather than generalized fecal contamination. Similarly, chloride exceedances were localized and parameter-specific, indicating that chemical non-compliance was not widespread across the investigated hotels. These findings support an integrated water safety approach in which *Legionella* results are interpreted together with general microbiological quality, operational monitoring, hydraulic conditions, and maintenance practices, rather than as an isolated endpoint [73,74,75,76].

The generalizability of these findings should be considered in relation to the regional and operational context of the study. Crete is a high-volume Mediterranean tourist destination with seasonal accommodation use, complex hotel water systems, and a regionalized public-health response structure. Therefore, the findings are most directly applicable to settings with similar tourism intensity, seasonal hotel operation, climate-sensitive water-system conditions, and reliance on regional environmental health services for TALD response. The microbiological positivity estimates should not be generalized to all hotels or accommodation facilities, because sampling was triggered by confirmed TALD notifications and was targeted toward implicated facilities and exposure-relevant sampling points. However, the operational lessons regarding notification delay, field-response coordination, repeated sampling, and integration of microbiological and operational indicators may be relevant to other Greek regions and Mediterranean tourist destinations facing similar TALD investigation challenges.

Several limitations should be acknowledged. First, the date on which the initial international notification reached the NPHO was not accessible to the regional investigating authorities. Furthermore, the accuracy of symptom onset dates relied on patient-reported information as recorded in notification forms and could not always be verified against clinical records. Minor inaccuracies in onset dates may affect the calculated values of RD, TDS, and OELR, and should be considered when interpreting these operational indicators. Therefore, the study could not separate delays occurring before NPHO receipt from delays between national and regional notification. The reporting-delay indicators reflect the interval from symptom onset to receipt of notification by the Regional Public Health Directorate, not the full international surveillance pathway. Second, sampling was intentionally targeted toward the patient’s room, distal outlets, and aerosol-generating points such as showers, reflecting the public-health purpose of TALD investigations. Some investigations may also have occurred after corrective actions had already started, which could have reduced the probability of detecting culturable *L. pneumophila*. Therefore, sample positivity estimates should be interpreted as findings from targeted exposure-driven investigation sampling and not as prevalence estimates for the entire hotel water system. Third, although environmental culture and serogrouping were performed, no clinical isolates were available for molecular comparison with the recovered environmental strains. Consequently, our findings cannot establish definitive source attribution or confirm transmission from a specific environmental source. Instead, this environmental data should be interpreted as a tool to support targeted risk assessment, source investigation, and public health decision-making for the implicated accommodation facilities. Fourth, some analyses were based on small numbers or selected subsets, particularly those involving indicator bacteriology, circulation-related parameters, and cross-regional investigations. Fifth, while multivariable logistic regression was used to adjust for potential confounders, the available software constraints and the limited number of implicated hotels prevented the reliable use of mixed-effects models to fully account for data non-independence. Therefore, the non-independence of samples collected from the same hotel networks could not be fully accounted for, which may affect the precision of the statistical inference and should be considered when interpreting the specific effect sizes.

Despite these limitations, this study provides a practical framework for evaluating TALD environmental investigations beyond simple positivity rates. By linking microbiological findings with notification timelines, field response, inspector capacity, and regional coordination, it shows where delays occur and how they may influence the timeliness and interpretability of environmental risk assessment. This approach can support faster notification pathways, better preparedness for field investigations, and more cautious interpretation of negative or discordant environmental results.

## 5. Conclusions

This study shows that TALD environmental investigations in hotel water systems require interpretation across three connected levels: microbiological evidence, water-system control, and operational timeliness. *L. pneumophila* was the dominant microbiological finding among positive samples, with SG2–15 representing the main serogroup category and SG1 remaining important for clinical interpretation, environmental risk assessment, and source investigation.

The findings support the importance of maintaining adequate disinfectant residuals, ensuring effective hot-water temperature control, and strengthening routine water safety management in tourist accommodation facilities. Negative cultures after delayed investigation should be interpreted cautiously, particularly when hotel operators have already implemented remedial measures before official sampling. In such cases, microbiological results should be assessed together with notification delay, sampling delay, corrective actions, and water-system risk factors.

The study also highlights the need for stronger coordination in multi-hotel and cross-regional TALD investigations. Central coordination by the Regional Public Health Directorate can reduce fragmentation, preserve epidemiological linkages, and support more consistent investigation and reporting. Future TALD response should prioritize faster notification to regional services, structured recording of operational timelines, repeated sampling when indicated, and molecular comparison between clinical and environmental isolates whenever possible. These measures would improve environmental risk assessment and strengthen source investigation, reduce false reassurance from delayed negative cultures, and support more timely public health action in TALD.

## Figures and Tables

**Table 1:** Distribution of analyzed environmental samples by sample type.

Sample Type	Frequency	Percent	Cum. Percent	Wilson 95% CL
Beach shower water	2	0.39%	0.39%	0.11–1.40%
Boiler outlet water	26	5.04%	5.43%	3.46–7.28%
Boiler return water	19	3.68%	9.11%	2.37–5.68%
Fountain water	2	0.39%	9.50%	0.11–1.40%
Garden irrigation water	5	0.97%	10.47%	0.41–2.25%
Garden shower water	2	0.39%	10.85%	0.11–1.40%
Garden tap water	2	0.39%	11.24%	0.11–1.40%
Ice machine water	1	0.19%	11.43%	0.03–1.09%
Jacuzzi pool water	7	1.36%	12.79%	0.66–2.77%
Jacuzzi shower water	4	0.78%	13.57%	0.30–1.98%
Kitchen sink tap water	12	2.33%	15.89%	1.34–4.02%
Municipal water inlet	15	2.91%	18.80%	1.77–4.74%
Pool shower water	28	5.43%	24.22%	3.78–7.73%
Pool water	9	1.74%	25.97%	0.92–3.28%
Public restroom tap water	5	0.97%	26.94%	0.41–2.25%
Public tap water	15	2.91%	29.84%	1.77–4.74%
Reclaimed water	1	0.19%	30.04%	0.03–1.09%
Restaurant tap water	3	0.58%	30.62%	0.20–1.70%
Room shower water	331	64.15%	94.77%	59.92–68.17%
Solar heater outlet water	2	0.39%	95.16%	0.11–1.40%
Spa pool water	1	0.19%	95.35%	0.03–1.09%
Spa shower water	13	2.52%	97.87%	1.48–4.26%
Water tank outlet	11	2.13%	100.00%	1.19–3.78%
Total	516	100.00%	100.00%	

Note: Percentages were calculated using all 516 collected environmental samples as the denominator. Thirteen samples could not be analyzed because of excessive background microbial growth; this analytical-status classification does not alter the sampling-point distribution shown here. CI, confidence interval.

**Table 2:** Definitions and public-health rationale of operational indicators used in TALD environmental investigations.

Indicator	Equation	Definition/Calculation Basis	Unit	Public Health Rationale
Reporting delay (RD)	Equation (1)	Interval from reported symptom onset date to receipt of official notification by the Regional Public Health Directorate	Days	Captures delay before activation of the regional environmental response
Time-to-first sampling (TTF)	Equation (2)	Interval from receipt of official notification by the Regional Public Health Directorate to first environmental sampling visit	Days	Measures field-response timeliness after notification
Time from symptom onset to first sampling (TDS)	Equation (3)	Interval from reported symptom onset date to first environmental sampling visit	Days	Captures total delay from illness onset to environmental investigation
Onset-to-first laboratory result (OELR)	Equation (4)	Interval from reported symptom onset date to availability of first environmental laboratory result	Days	Measures total delay until microbiological information becomes available
Notification-to-first laboratory result (NELR)	Equation (5)	Interval from receipt of official notification by the Regional Public Health Directorate to availability of first environmental laboratory result	Days	Measures post-notification time to actionable laboratory information
Efficiency ratio (ER)	Equation (6)	14-day operational reference benchmark divided by TTF	Ratio	Describes whether first sampling was initiated faster or slower than the two-week operational reference
Sampling productivity (SPD)	Equation (7)	Number of samples collected during the first sampling visit divided by TTF	Samples/day	Describes sampling output relative to response delay
Service-adjusted sampling efficiency (SASE)	Equation (8)	Sampling productivity adjusted for inspector availability	Composite	Describes service-level sampling efficiency while accounting for staffing differences
Positivity rate	Equation (9)	Number of analyzed samples positive for *L. pneumophila* ≥ 50 CFU/L divided by the total number of analyzed samples	%	Summarizes microbiological detection at or above the analytical detection limit
Threshold exceedance rate	Equation (10)	Number of analyzed samples with *L. pneumophila* > 1000 CFU/L divided by the total number of analyzed samples	%	Identifies higher-level contamination with greater operational relevance for corrective action
SG1 proportion	Equation (11)	Number of *L. pneumophila* serogroup 1 positive samples divided by the number of serotyped *L. pneumophila* positive samples	%	Captures the proportion of the serogroup most strongly associated with human LD
Regional notification delay (RND)	Equation (12)	Interval from reported symptom onset date to receipt of notification by the competent Regional Unit	Days	Captures regional notification delay in cross-regional investigations
Regional time-to-first sampling (TTF_r_)	Equation (13)	Interval from receipt of notification by the competent Regional Unit to first sampling visit in that Regional Unit	Days	Measures local field-response timeliness in cross-regional investigations
Regional efficiency ratio (ER_r_)	Equation (14)	14-day operational reference benchmark divided by regional TTF	Ratio	Describes regional timeliness relative to the two-week operational reference
Regional service-adjusted sampling efficiency (SASE_r_)	Equation (15)	Regional sampling productivity adjusted for regional inspector availability	Composite	Supports comparison of field-response performance between Regional Units with different inspector capacity

Note: ER, SPD, SASE, and their regional equivalents are descriptive operational performance indicators. They were used to compare timeliness, sampling effort, and service capacity within the TALD investigation pathway and should not be interpreted as externally validated epidemiological risk scores. The 14-day reference was used as an operational benchmark derived from the ELDSNet/EWGLI two-week timeframe for preliminary risk assessment and initiation of control measures after an alert, rather than as a strict microbiological sampling deadline.

**Table 3:** Operational and microbiological performance indicators for completed TALD environmental investigations in Crete, 2025.

Indicator	Equation	Median (IQR)	Range	*n*	Unit
RD	(1)	31.0 (26.5–42.3)	1–103	20	days
TTF	(2)	14.5 (4.8–38.3)	1–182	20	days
TDS	(3)	47.5 (38.5–91.5)	12–235	20	days
OELR	(4)	67.0 (50.5–110.5)	24–252	20	days
NELR	(5)	29.0 (22.3–59.3)	13–199	20	days
ER	(6)	0.97 (0.37–2.97)	0.08–14.00	20	ratio
SPD	(7)	0.49 (0.16–1.85)	0.02–10.50	20	samples/day
SASE	(8)	0.18 (0.07–0.73)	0.01–2.63	20	composite
Positivity rate	(9)	21.5 (8.6–48.3)	0.0–100.0	20	%
Threshold exceedance rate	(10)	1.4 (0.0–17.3)	0.0–100.0	20	%
SG1 proportion	(11)	10.0 (0.0–50.0)	0.0–100.0	16	%

Note: Data are medians with interquartile ranges (IQRs) and ranges from completed hotel–sampling-round investigations with recorded sample counts (*n* = 20). Equation numbers correspond to the operational indicators defined in Section 2.5 and summarized in Table 2. RD, reporting delay; TTF, time-to-first sampling; TDS, time from symptom onset to first sampling; OELR, onset-to-first laboratory result; NELR, notification-to-first laboratory result; ER, efficiency ratio; SPD, sampling productivity; SASE, service-adjusted sampling efficiency.

**Table 4:** Exploratory comparison of reporting-delay category and environmental *L. pneumophila* detection among completed investigations.

Outcome	RD Category	Positive	Negative	Total	RR (Delayed vs. Timely)	95% CI	Fisher’s *p*-Value
*L. pneumophila* ≥ 50 CFU/L	RD ≤ 14 days	2	0	2	—	—	—
*L. pneumophila* ≥ 50 CFU/L	RD > 14 days	14	4	18	0.78	0.61–1.00	1.00
*L. pneumophila* > 1000 CFU/L	RD ≤ 14 days	1	1	2	—	—	—
*L. pneumophila* > 1000 CFU/L	RD > 14 days	9	9	18	1.00	0.23–4.31	1.00

Note: Analyses were restricted to completed investigations with recorded sample counts (*n* = 20). Timely reporting was defined as RD ≤ 14 days. RR compares delayed versus timely reporting; Fisher’s exact test was used because of small cell counts. This comparison was exploratory. Reporting-delay category was not statistically associated with *L. pneumophila* detection in this dataset.

**Table 5:** Operational and microbiological indicators for cross-regional TALD investigations, Crete, 2025.

Regional Unit	RND (Days)	TTF_r_ (Days)	ER_r_	SASE_r_	cTTF_r_ (Days)	cER_r_	cSASE_r_	SLR
A	25–49	37–88	0.16–0.38	0.04–0.06	18.5–44.0	0.32–0.76	0.15–0.24	0.50
B	49	42	0.33	0.18	42.0	0.33	0.18	1.00
C	31	15–18	0.78–0.93	0.07–0.29	3.8–4.5	3.11–3.73	1.11–4.67	0.25
D	41	27	0.52	0.17	27.0	0.52	0.17	1.00

Note: Values are presented as ranges when more than one completed regional investigation was available. TTF_r_, ER_r_, SASE_r_, cTTF_r_, cER_r_, and cSASE_r_ were calculated only for completed regional investigations. RND, regional notification delay; TTF_r_, regional time-to-first sampling; ER_r_, regional efficiency ratio; SASE_r_, regional service-adjusted sampling efficiency; SLR, service load ratio.

**Table 6:** Recommended inspectors dedicated to TALD environmental inspections and first-round sampling by Regional Unit to achieve TTF ≤ 14 days under current workload.

Regional Unit	Observed Inspectors Available	Median TTF_r_ (Days)	Capacity-Only Estimate, Inspectors	Suggested TALD-Dedicated Inspector Capacity
A	4	37.0	11	6–8
B	2	23.5	4	3–4
C	8	4.0	3	3–4
D	2	13.0	2	2

Note: Capacity estimates assume delays depend only on staffing; suggested capacity also accounts for operational improvements. TTF_r_: regional time-to-first sampling.

**Table 7:** Multivariable logistic regression analysis for facility and water-system characteristics associated with *L. pneumophila* detection.

Variable	aOR	95% CI	*p*-Value	E-Value (Lower Bound)
Free chlorine < 0.2 mg/L	3.11	1.14–8.51	0.027	5.67 (1.54)
Hot water temperature < 55 °C	5.47	1.98–15.08	0.001	10.42 (3.37)
Hotel rating < 4 stars	4.95	2.06–11.89	<0.001	9.38 (3.54)
Hotel size > 200 beds	0.26	0.13–0.51	<0.001	—

Note: aOR, adjusted odds ratio; CI, confidence interval. Non-applicable or missing indicator categories were included in the model to retain observations, but are not shown because they do not represent interpretable exposure effects. Because multiple samples originated from the same hotels and clustering could not be fully accounted for, these estimates should be interpreted cautiously with respect to precision and should not be considered causal effects.

**Table 8:** Positivity of *L. pneumophila* serogroups by sampling round.

*L. pneumophila* Serogroup	First Sampling Round, *n* = 253	Second Sampling Round, *n* = 129	Third Sampling Round, *n* = 61
SG1	18 (7.11%; 95% CI: 4.27–11.01)	12 (9.30%; 95% CI: 4.90–15.69)	1 (1.64%; 95% CI: 0.04–8.80)
SG2	5 (1.98%; 95% CI: 0.64–4.55)	4 (3.10%; 95% CI: 0.85–7.75)	0 (0.00%; 95% CI: 0.00–5.87)
SG3	17 (6.72%; 95% CI: 3.96–10.54)	3 (2.33%; 95% CI: 0.48–6.65)	1 (1.64%; 95% CI: 0.04–8.80)
SG4	0 (0.00%; 95% CI: 0.00–1.45)	0 (0.00%; 95% CI: 0.00–2.82)	0 (0.00%; 95% CI: 0.00–5.87)
SG5	0 (0.00%; 95% CI: 0.00–1.45)	0 (0.00%; 95% CI: 0.00–2.82)	0 (0.00%; 95% CI: 0.00–5.87)
SG6	12 (4.74%; 95% CI: 2.47–8.14)	12 (9.30%; 95% CI: 4.90–15.69)	1 (1.64%; 95% CI: 0.04–8.80)
SG7	0 (0.00%; 95% CI: 0.00–1.45)	1 (0.78%; 95% CI: 0.02–4.24)	0 (0.00%; 95% CI: 0.00–5.87)
SG8	14 (5.53%; 95% CI: 3.06–9.11)	16 (12.40%; 95% CI: 7.26–19.36)	2 (3.28%; 95% CI: 0.40–11.35)
SG9	10 (3.95%; 95% CI: 1.91–7.15)	0 (0.00%; 95% CI: 0.00–2.82)	0 (0.00%; 95% CI: 0.00–5.87)
SG10	0 (0.00%; 95% CI: 0.00–1.45)	0 (0.00%; 95% CI: 0.00–2.82)	0 (0.00%; 95% CI: 0.00–5.87)
SG11	0 (0.00%; 95% CI: 0.00–1.45)	0 (0.00%; 95% CI: 0.00–2.82)	0 (0.00%; 95% CI: 0.00–5.87)
SG12	0 (0.00%; 95% CI: 0.00–1.45)	0 (0.00%; 95% CI: 0.00–2.82)	0 (0.00%; 95% CI: 0.00–5.87)
SG13	0 (0.00%; 95% CI: 0.00–1.45)	0 (0.00%; 95% CI: 0.00–2.82)	0 (0.00%; 95% CI: 0.00–5.87)
SG14	0 (0.00%; 95% CI: 0.00–1.45)	0 (0.00%; 95% CI: 0.00–2.82)	0 (0.00%; 95% CI: 0.00–5.87)
SG15	1 (0.40%; 95% CI: 0.01–2.18)	0 (0.00%; 95% CI: 0.00–2.82)	1 (1.64%; 95% CI: 0.04–8.80)
SG2–15	57 (22.53%; 95% CI: 17.53–28.18)	34 (26.36%; 95% CI: 18.99–34.84)	4 (6.56%; 95% CI: 1.82–15.95)

Note: Values are positive samples with percentages and exact 95% confidence intervals within each sampling round. SG2–15 denotes isolates recorded collectively as serogroups 2–15.

**Table 9:** Microbiological non-compliance among selected water samples with detailed indicator bacteriology.

Parameter	Non-Compliant/Total	% Non-Compliant	95% CI (Wilson)
Total coliforms	11/23	47.83%	29.17–67.05%
*E. coli*	0/23	0.00%	0.00–14.83%
Enterococci	1/23	4.35%	0.77–21.41%
Free chlorine	2/13	15.38%	4.33–42.21%

Note: CI, confidence interval.

## Data Availability

The data presented in this study are available on request from the corresponding author due to privacy and confidentiality restrictions.

## Abbreviations

The following abbreviations are used in this manuscript:

aOR	Adjusted odds ratio
APHA	American Public Health Association
BCYE	Buffered charcoal yeast extract
cER_r_	capacity-normalized regional efficiency ratio
CFU	Colony-forming units
CI	Confidence interval
cSASE_r_	capacity-normalized regional service-adjusted sampling efficiency
cTTF_r_	capacity-normalized regional time-to-first sampling
*E. coli*	*Escherichia coli*
ECDC	European Centre for Disease Prevention and Control
ELDSNet	European Legionnaires’ Disease Surveillance Network
ELSTAT	Hellenic Statistical Authority
EQA	External Quality Assessment
ER	Efficiency ratio
ER_r_	Regional efficiency ratio
EURL-PH-LEGI	European Reference Laboratory in Public Health on Legionella
E-value	Sensitivity analysis value for unmeasured confounding
EWGLI	European Working Group for Legionella Infections
GVPC	Glycine, vancomycin, polymyxin B, and cycloheximide agar
HAS	Hellenic Accreditation System
HCl	Hydrochloric acid
ID-TTF	inspector-days to first sampling
IQR	Interquartile range
ISO	International Organization for Standardization
LCL	lower confidence limit
LD	Legionnaires’ disease
NELR	Notification-to-first laboratory result
NPHO	National Public Health Organization
OELR	Onset-to-first laboratory result
PCR	Polymerase chain reaction
RD	Reporting delay
RND	Regional notification delay
RR	Risk ratio
SASE	Service-adjusted sampling efficiency
SASE_r_	Regional service-adjusted sampling efficiency
SG1	Serogroup 1
SG2–15	Serogroups 2–15
SLR	Service load ratio
SPD	Sampling productivity
TALD	Travel-associated Legionnaires’ disease
TDS	Time from symptom onset to first sampling
TTF	Time-to-first sampling
TTF_r_	Regional time-to-first sampling
UAT	Urinary antigen test
UCL	upper confidence limit
UKNEQAS	United Kingdom National External Quality Assessment Service
WHO	World Health Organization
